# Percutaneous transforaminal endoscopic discectomy for Upper Lumbar Disc Herniation versus lower lumbar disc herniation: clinical outcomes and technical consideration

**DOI:** 10.1186/s12891-024-07588-7

**Published:** 2024-06-15

**Authors:** Shuo Yuan, Yuqi Mei, Lei Zang, Xuanyu Lu, Ning Fan, Peng Du

**Affiliations:** Department of Orthopedics, Capital Medical University, Beijing Chaoyang Hospital, 5 JingYuan Road, Shijingshan District, Beijing, 100043 China

**Keywords:** Upper lumbar disc herniation, Treatment outcomes, Discectomy, Percutaneous transforaminal endoscopic discectomy

## Abstract

**Background:**

Upper lumbar disc herniation (ULDH) accounts for 1-10% of all lumbar disc herniations (LDH). This study aimed to evaluate the clinical characteristics and outcomes of patients with ULDH who underwent percutaneous transforaminal endoscopic discectomy (PTED) compared with those with lower LDH.

**Methods:**

60 patients with ULDH or L4–L5 LDH treated with PTED between May 2016 and October 2021. MacNab criteria, visual analog scale (VAS) of back pain and leg pain, and Japanese Orthopedic Association (JOA) were evaluated before and after surgery.

**Results:**

In the L1–L3 group, 59.1% of the patients had a positive femoral nerve tension test, and 81.8% of the patients had a sensory deficit. Both groups showed significant improvements in VAS scores for low back and leg pain, and JOA scores postoperatively. No significant differences in the degree of improvement were observed between the two groups. The excellent/good rate was 81.8% in the L1–L3 group and 84.2% in the L4–L5 group, showing no significant difference.

**Conclusion:**

PTED has comparable efficacy in treating ULDH as it does in treating lower LDH, it is a safe and effective treatment method for ULDH.

## Background

Lumbar disc herniation (LDH) is the most common spinal disorder and the most common indication for spinal surgery [1]. Upper lumbar disc herniation (ULDH) accounts for 1–10% of all LDH [2–7]. The unclear definition of ULDH is a reason for the wide variation in its incidence. Some authors considered the upper lumbar discs to be located at L1–L2 or L2–L3 [2, 4, 5, 8–10]. Other authors expanded the definition to T12–L1 and L3–L4 [3, 6, 7, 11, 12]. Upper lumbar discs were defined as L1–L2 and L2–L3 levels due to the specific anatomical characteristics and less favorable clinical outcome of L1–L2 and L2–L3 levels of LDH [4, 13]. 

Spinal canals in the upper lumbar area are narrower and contain multiple nerve roots, cauda equina, and conus medullaris compared with those in the lower lumbar area. Additionally, the length of the lamina and the width of the isthmus are shorter than those in the lower lumbar area [11, 14]. Because of these anatomical factors, surgery has a less favorable outcomes for ULDH than for lower LDH [4]. Therefore, the choice of surgical approach is crucial.

Recently, many studies have shown that percutaneous transforaminal endoscopic discectomy (PTED) can achieve the same clinical outcomes as conventional open surgery [15]. Compared with traditional open decompression and fusion surgery, PTED has several advantages, such as less impact on segmental stability, shorter hospital stays, and being performed under local anesthesia [16, 17]. Additionally, conventional open surgery has a high risk of iatrogenic isthmic fracture due to the unique anatomical characteristics of the upper lumbar area [11]. With the development of surgical techniques and instruments, PTED has been considered an alternative method for ULDH. Because the foramen size in the upper lumbar area is larger, it is easier for a transforaminal endoscope to access the upper portion of the foramen than the lower lumbar area. Therefore, it is easier to remove herniated discs using a transforaminal endoscope. Studies on PTED for ULDH are limited, and there are even fewer studies that have compared the clinical outcomes between patients with ULDH and those with lower LDH treated with PTED.

This retrospective comparative study aimed to evaluate the clinical characteristics and outcomes of patients with LDH at the L1–L2 and L2–L3 levels who underwent PTED, compared with those presenting with LDH at the L4–L5 level, in order to demonstrate the comparable efficacy of PTED for both conditions. Because the foramen at L4–L5 is not obstructed by the iliac crest, a standard puncture angle can be ensured. Furthermore, the technical strategies and considerations of PTED for ULDH were investigated.

## Materials and methods

### Patients and data Collection

This study was approved by the Ethics Committee of Beijing Chaoyang Hospital (registration number: 2021-KE-298) and was conducted in accordance with the guidelines of the Declaration of Helsinki. A total of 22 consecutive patients with ULDH treated with PTED between May 2016 and October 2021 at our center. Stratified sampling was conducted for patients admitted with L4-L5 LDH during the same period, based on the proportional annual admission rates of patients with ULDH. Ultimately, a sample of 38 patients was selected. The inclusion criteria included (1) patients with soft disc herniation at the L1–L2, the L2–L3 or the L4–L5 levels as demonstrated by computed tomography (CT) and magnetic resonance imaging (MRI); (2) failure of conservative treatment for at least 3 months and (3) no evidence of segment instability on plain radiography. The exclusion criteria included (1) patients with disc herniation at the L3–L4 or the L5–S1 levels, (2) history of lumbar spine surgery on the same level, (3) spondylolisthesis > grade I, (4) spinal tumor or infection, and scoliosis, and (5) patients with incomplete information during follow up.

Demographic and clinical data of the patients, including age, sex, duration of symptoms (DOS), body mass index (BMI), clinical signs, and duration of follow-up, were collected from medical records. The anatomic location and migrated direction of disc herniation was obtained based on the MRI.

### Surgical intervention

All procedures were performed in a single foramen. PTED was performed as follows: the patient was placed in a prone position, and the whole procedure was performed under local anesthesia. The entry point was established 8–10 cm lateral to the spinal midline at the L1–L3 level and 10–12 cm lateral to the spinal midline at the L4–L5 level. Initially, a spinal needle was inserted into the facet joint and subsequently replaced with a guidewire. Then, an 8 mm working cannula was placed after expanding the surgical approach with serial hollow tapered cannulas. Next, a part of the facet was removed using a burr saw to access the intraforaminal area. If necessary, the resection range was enlarged to the medial side of the superior or partial inferior articular process. Decompression was performed using continuous irrigation under direct vision. Osteophytes, hyperplasia of the ligamentum flavum, perineural fat, and bulging of the disk surface were removed. The entire exiting nerve root was carefully probed to ensure complete decompression. Early ambulation was permitted 1 day after surgery with the protection of waist support. Full activity was allowed 1 month after surgery.

**Fig. 1:**
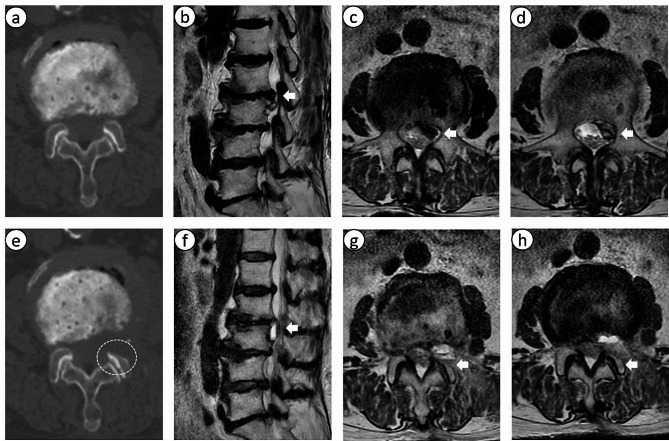
Images from a typical case of percutaneous endoscopic transforaminal discectomy in a 74-year-old female with upper lumbar disc herniation at L2–3. (a) Preoperative axial CT. (b, c, d) Preoperative sagittal and axial T2-weighted MRI shows the left side lumbar disc herniation at L2–3. (e) Postoperative axial CT shows partial removal of the superior articular process. (f, g, h) Postoperative sagittal and axial T2-weighted MRI illustrates complete excision of the prolapsed disc, without residual disc at L2–3

**Fig. 2:**
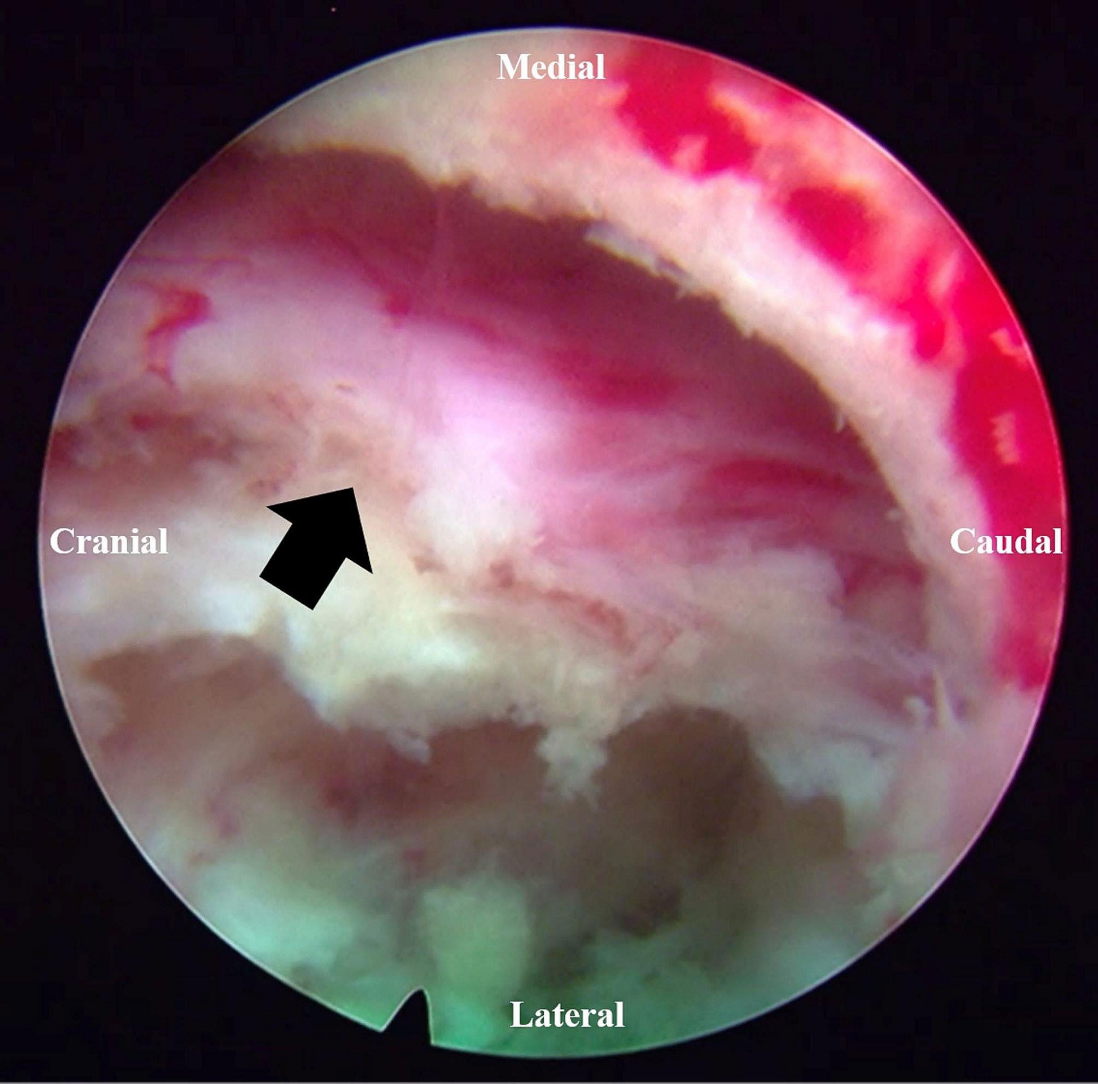
Intraoperative view of the L3 nerve root after decompression. The black arrow indicates the L3 nerve root

A representative case of a patient who successfully underwent PTED is shown in Figs. 1 and 2.

### Clinical Assessment and follow-up

Surgical parameters, including surgical time, estimated blood loss and postoperative hospitalization stay, complications, and recurrence, were obtained from the surgical records.

Visual analog scale (VAS) scores for low back and leg pain, Japanese Orthopedic Association Scores (JOA) scores, and modified MacNab criteria were evaluated for clinical outcome assessment. Patients were asked to complete the assessment before and after the surgery, and the final follow-up was accomplished in October 2023. All eligible patients were followed up for at least two years.

### Statistical analysis

Statistical analyses were performed using SPSS 24.0 (IBM Corp., Armonk, NY, USA). Continuous variables were analyzed using the independent sample *t*-test, whereas categorical variables were analyzed using the chi-square test. A *P*-value < 0.05 was considered statistically significant.

## Results

A total of 60 eligible patients were included in this study, with a mean follow-up period of 52.6 ± 18.7 months and a minimum follow-up period of 24 months. The L1–L2 and L2–L3 group (L1–L3 group) included 22 patients diagnosed with ULDH (4 with LDH at the L1–L2 level and 18 at the L2–L3 level). Significant differences in positive femoral nerve tension test and sensory deficit were observed between the two groups. However, no significant differences in positive Lasègue sign and motor deficit were observed. No significant differences in age, sex, DOS, BMI, and location of disc herniation were observed between the groups. Table 1 shows detailed information about the patients (Table 1).

**Table 1:** Demographic and clinical information of patients

Parameter	L1–L3	L4–L5	*P* value
Number of patients	22	38	
Average ages (yr)	65.1 ± 13.3	65.0 ± 11.9	0.862
Age range (yr)	28–79	35–83	
Sex (male/female)	14/8	17/21	0.158
Duration of symptoms (months)	73.6 ± 50.2	70.7 ± 62.9	0.905
Body mass index	26.1 ± 4.5	26.7 ± 4.9	0.649
Clinical signs			
Lasègue sign+	8(36.4%)	13(34.2%)	0.866
Femoral nerve tension test +	13(59.1%)	1(2.6%)	0.003*
Motor deficit	3(13.6%)	11(28.9%)	0.177
Sensory deficit	18(81.8%)	19(50.0%)	0.015*
Location of disc herniation			0.936
Central	6(27.3%)	12(31.6%)	
Parainforaminal	14(63.6%)	23(60.5%)	
Intraforaminal	2(9.1%)	3(7.9%)	
Follow-up periods(months)	47.7 ± 14.9	55.5 ± 20.2	0.091

Results are the mean ± standard deviation; *: significant difference between L1–L3 group and L4–L5 group, *P* < 0.05

Table 2 shows a comparison of surgical data between the two groups. (Table 2) All patients underwent successful PTED. The L1–L3 group showed a trend toward longer surgical time, longer postoperative hospitalization stays, and more estimated blood loss than the L4–L5 group, but without statistical significance. No complications, such as infections, deep vein thromboses, or dural tears, were observed in either group.

**Table 2:** Comparison of operation parameters between the two groups

Parameter	L1–L3	L4–L5	*P* value
Operation time (min)	104.6 ± 27.1	98.0 ± 31.7	0.329
Estimated blood loss(ml)	18.2 ± 3.9	15.4 ± 4.7	0.635
Postoperative hospitalization stay	6.3 ± 3.5	5.4 ± 2.9	0.925

Results are the mean ± standard deviation;

The patients were followed up to obtain measures of clinical outcomes (Table 3). Significant differences in the preoperative VAS score for low back pain were observed between the two groups. No significant difference was found in preoperative leg pain VAS scores, JOA scores. The VAS scores for low back pain, the VAS scores for leg pain and JOA scores improved significantly in both groups after surgery. However, no significant difference in the improvement in all the assessments was observed between the two groups.

**Table 3:** Preoperative and follow-up VAS, JOA scores and Macnab criteria assessment

	L1–L3	L4–L5	*P* value
VAS of low back pain			
Preoperative	64.5 ± 15.4	56.4 ± 8.9	0.015*
Postoperative	24.6 ± 20.0^#^	21.9 ± 17.3^#^	0.781
Final follow-up	22.7 ± 19.9^#^	20.3 ± 17.7^#^	0.929
Improvement	41.8 ± 19.4	38.1 ± 19.6	0.782
VAS of leg pain			
Preoperative	68.9 ± 14.3	71.6 ± 10.6	0.143
Postoperative	25.6 ± 20.4^#^	23.5 ± 18.5^#^	0.906
Final follow-up	24.5 ± 20.6^#^	21.8 ± 19.2^#^	0.924
Improvement	44.4 ± 21.7	49.7 ± 20.8	0.677
JOA scores			
Preoperative	9.3 ± 2.9	11.7 ± 3.3	0.830
Postoperative	19.7 ± 5.4^#^	18.7 ± 4.7^#^	0.570
Final follow-up	21.8 ± 6.0^#^	21.1 ± 5.5^#^	0.860
Improvement	12.5 ± 5.7	9.3 ± 5.1	0.908
Macnab criteria assessment			0.324
Excellence	13(59.1%)	21(55.3%)	
Good	5(22.7%)	11(28.9%)	
Fair	3(16.7%)	4(10.5%)	
Poor	1(5.6%)	2(5.3%)	
Excellence/good rate	81.8%	84.2%	

Results are the mean ± standard deviation; *: significant difference between L1–L3 group and L4–L5 group, *P* < 0.05; ^#^: significant difference between preoperative group and postoperative or final follow up group, *P* < 0.05

No significant differences in the distribution of the MacNab criterion assessment were observed between the two groups (*P* = 0.641) (Table 3). The excellent/good rate was 81.8% in the L1–L3 group and 84.2% in the L4–L5 group, showing no significant difference, which suggests similar clinical effectiveness achieved in both groups.

## Discussion

The incidence of ULDH is much lower than that of lower LDH. Thus, ULDH has received little attention and has a high misdiagnosis rate [18]. Conventional open surgery was considered the gold standard for treating of ULDH [19]. With the development of surgical techniques and instruments, PTED has become an alternative method for ULDH and has achieved clinical outcomes comparable to conventional open surgery [7]. 

The symptoms and signs of ULDH differ from those of lower LDH due to the anatomical characteristics of the upper lumbar spine. Compared with the spinal canals in the lower lumbar spine, those in the upper lumbar area are narrower and contain multiple nerve roots, cauda equina and conus medullaris [11, 14]. In patients with ULDH, the Lasègue sign is usually normal, whereas the femoral nerve tension test is positive in 84–94% of those with ULDH [14, 20]. Because the femoral nerve is primarily composed of L2–L4 nerve roots, the pain provoked by the femoral nerve stretch test is believed to be caused by stretching of the femoral nerve [21]. Therefore, patients with ULDH are more likely to show positive results on the femoral nerve stretch test. Kido et al. reported that the sensory deficit localized in the thigh area proximal to the knee joint is a specific sign of L2 nerve root disturbance [22]. In this study, the femoral nerve tension test was positive in 59.1% of the patients in the L1–L3 group compared with 2.6% in the L4-L5 group. Furthermore, the sensory deficit was found in 81.8% of the patients in the L1–L3 group and 50.0% in the L4–L5 group. Albert et al. suggested that the lack of specificity in the clinical signs of ULDH is attributed to the presence of cross-innervation between spinal nerves in the upper lumbar area [21]. 

PTED has unique advantages over other surgical procedures for treating ULDH. The foramen of the upper lumbar spine is relatively larger than that of the lower levels, allowing surgical instruments to reach the herniated disc directly without foraminoplasty or with the need to perform small-scale foraminoplasty. Furthermore, by approaching through the foramen, the bony structures and ligamentum flavum are not disrupted, minimizing the impact on spinal stability and reducing disturbance to the neural tissues within the spinal canal. Additionally, PTED can be performed under local anesthesia allowing the patient to remain conscious during the operation to avoid accidental nerve damage by the surgeon. Li et al. showed that PTED achieved a similar effect to conventional surgical method but significantly reduced the surgical time, blood loss, postoperative hospitalization time, and incidence of wound complication [7]. Jiang et al. proposed that applying unilateral biportal endoscopic discectomy for treating LDH demonstrated similar clinical outcomes to PTED [23]. Unilateral biportal endoscopic discectomy is also a minimally invasive procedure, but it primarily decompresses the dorsal side, which involves ipsilateral laminotomy and contralateral sublaminotomy [24]. Since the length of the lamina and the width of the isthmus are short, and the inferior edge of the lamina obscures the anterior intervertebral disc in the upper lumbar area, a part of the lamina had to be removed during the posterior approach procedure, thereby increasing the risk of isthmus injury, which in turn leads to lumbar instability.

Because ULDH has a low incidence and anatomical complexity, more surgical experience and accurate dissection are required during the operation. In this study, the surgical time for ULDH was slightly longer than that for lower LDH, but the difference is not significant (*P* > 0.05). Based on the MacNab criteria, the outcomes were excellent/good in 81.8% of patients in the L1–L3 group and 81.6% in the L4-L5 group. The VAS scores for low back pain and leg pain and JOA scores at the final follow-up were significantly improved in both groups, without significant difference between two groups. The study findings suggest that PTED can significantly improve L1–L3 disc herniation to the same degree as that for L4-L5 disc herniation.

PTED has a low complication rate in the treatment of ULDH. According to previous studies, common complications include cerebrospinal fluid leakage, neural injury, and postoperative dysesthesia [25]. These can usually be cured with conservative treatment [7, 26]. Further technical points of PTED should be considered in the treatment of ULDH to obtain a better clinical outcome. First, the insertion angle for the needle and working sheath should be more acute when targeting an upper lumbar disc than the angle required for a disc at a lower level. This is because the disc surface of the upper lumbar discs is more concave and sharply angled in the axial plane. Thus, the horizontal approach carries a potential risk of dural sac injury. Second, the foramen is relatively large, and the dural sac is readily exposed through the foraminal window in the upper lumbar area. Hence, the annular puncture point should be more lateral [10]. 

This study has several limitations. First, this study was a retrospective study and the number of patients in the L1–L3 group was smaller than that in the L4-L5 group. Thus, further prospective multicenter studies with a large sample size are needed. Second, this study was based on clinical outcomes only. Future work requires comparison of radiologic outcomes further to evaluate the rate of facet preservation and the occurrence of iatrogenic complications such as spondylolysis.

## Conclusion

This study demonstrates that, despite the anatomical complexity of the upper lumbar area, PTED is equally effective in treating ULDH as it is in treating lower LDH. Therefore, PTED is a safe and effective treatment method for ULDH.

## Data Availability

The datasets generated during and/or analyzed during the current study are available from the corresponding author on reasonable request.

## Abbreviations

LDH: Lumbar disc herniation
ULDH: Upper lumbar disc herniation
PTED: Percutaneous transforaminal endoscopic discectomy
CT: Computed tomography
MRI: Magnetic resonance imaging
DOS: Duration of symptom
BMI: Body mass index
VAS: Visual analog scale
JOA: Japanese orthopedic association scores
